# HKSiamFC: Visual-Tracking Framework Using Prior Information Provided by Staple and Kalman Filter

**DOI:** 10.3390/s20072137

**Published:** 2020-04-10

**Authors:** Chenpu Li, Qianjian Xing, Zhenguo Ma

**Affiliations:** College of Biomedical Engineering and Instrument Science, Zhejiang University, Hangzhou 310027, China; 11715045@zju.edu.cn (C.L.); xingqianjian@zju.edu.cn (Q.X.)

**Keywords:** visual tracking, Staple, SiamFC, Kalman filter

## Abstract

In the field of visual tracking, trackers based on a convolutional neural network (CNN) have had significant achievements. The fully-convolutional Siamese (SiamFC) tracker is a typical representation of these CNN trackers and has attracted much attention. It models visual tracking as a similarity-learning problem. However, experiments showed that SiamFC was not so robust in some complex environments. This may be because the tracker lacked enough prior information about the target. Inspired by the key idea of a Staple tracker and Kalman filter, we constructed two more models to help compensate for SiamFC’s disadvantages. One model contained the target’s prior color information, and the other the target’s prior trajectory information. With these two models, we design a novel and robust tracking framework on the basis of SiamFC. We call it Histogram–Kalman SiamFC (HKSiamFC). We also evaluated HKSiamFC tracker’s performance on dataset of the online object tracking benchmark (OTB) and Temple Color (TC128), and it showed quite competitive performance when compared with the baseline tracker and several other state-of-the-art trackers.

## 1. Introduction

Visual tracking plays a fundamental role in the field of computer vision. It is widely used, including in auto-monitoring [1], autonomous driving [2], surveillance [3], robotics [4], and motion-based recognition [5]. The main task of visual tracking is distinguishing a target from complicated environments in video, and to provide an accurate result of the target size and location co-ordinates [6], usually with a bounding box. The only reference that the tracker can use is the labeled ground truth in the first frame. This seemingly easy task has met many difficulties. Lacking prior information about the target’s appearance, environment illumination changes [7,8,9], scene clutter [10], and many other factors can lead to the tracker drifting, and make tracking fail. To solve these challenging problems, a large variety of novel trackers have been proposed over the years. They are constructed on different theoretical bases and aimed at solving different challenges. However, all those trackers share the same key step: extracting powerful features from the target that are distinct enough to express target properties. Then, those features can be used as a reference to locate the right target and leave out extraneous distractors. 

Recently, deep learning (DL) has flourished and benefited many computer-vision-related tasks [11,12,13,14,15]. A convolutional neural network (CNN) [16] is a typical deep-learning architecture that is often used in image processing. Unlike traditional features, a CNN can use many training data and learn to capture target features from different levels. The number of levels is decided by the CNN’s number of layers, and each layer acts as a specific feature-pattern extractor. So, features extracted by CNN are very powerful and widely used by many trackers. 

Typically, Bertinetto designed a fully-convolutional Siamese (SiamFC) tracker [17] using Alexnet [18]. It achieved state-of-the-art performance on the visual-tracking benchmark, showing its significant potential in visual tracking. SiamFC models visual tracking as a similarity learning problem, and the CNN in SiamFC is trained end-to-end especially for visual tracking. This determines that the features it extracts are more powerful than other CNNs which are trained in other tasks. For example, the deep features used in many correlation filter trackers are extracted by CNNs which are trained in image detection task. 

However, there are two main disadvantages in the SiamFC tracking framework. First, it only uses the features in the last layer to generate the final response scores. Those high-level features are robust to the noise but lack detail information of the target, so they are not discriminative enough when the distractor belongs to the same category with the target. Second, the way to train the SiamFC determines that all the patches in the search region have the same contribution to the final response score map. So, when a distractor appears in the search region, no matter where its location is, it may generate a high response score and cause the tracking to fail. 

Our research focuses on addressing the problems listed above. We find that these two problems essentially show the same key point that SiamFC lacks enough prior information about the target. So our motivation is to provide prior information about the target to the original SiamFC tracker, containing appearance information and motion information. As for the first problem, the high-level features’ shortcoming can be complemented by adding more prior detail information about the target’s appearance, and we think color information is a good choice because color distribution of the target copes well with appearance variation. We analysis the histogram score model used in Staple and find it can be improved to achieve our goal here. The other problem can be improved by adding prior trajectory information of the target. Then, we can use the trajectory information to generate a Gaussian likelihood map. Then, the response score of the distractor far from the target can be suppressed by this map. The Kalman filter is a good choice for us to get the prior trajectory information we need. In this paper, we design a novel tracking framework based on the motivation above. The framework contains three main components, namely SiamFC, histogram score model, and Kalman filter model. This tracker is called Histogram–Kalman SiamFC (HKSiamFC). The main contributions of our contributions are summarized as follows:

(1) We improve the Staple’s histogram score model and introduce it into our HKSiamFC tracker. Unlike the Staple, we don’t use the histogram score model to generate an integral image, but optimize it with Gaussian filter and use it to provide appearance information of the target.
(2) We build a Gaussian likelihood map on the basis of a Kalman filter to describe the target’s trajectory information, and then use this Gaussian likelihood map and the histogram score map to process the SiamFC’s response score map by element-wise multiplication.
(3) We evaluated HKSiamFC tracker on the dataset of online tracking benchmark (OTB) and Temple Color (TC128), and experiments showed that it achieved competitive performance when compared with baseline trackers and other state-of-the-art trackers.

The rest of the paper is organized as follows. In Section 2, we introduce some related works in visual tracking. Then, we present our tracking framework in Section 3. In Section 4, we evaluate the performance of our tracker on the mainstream dataset in visual tracking and compare it with some state-of-the-art trackers. Section 5 presents a summary of our work. 

## 2. Related Work

### 2.1. Correlation-Filter-Based Trackers

Most correlation filter-based trackers have three characteristics in common: learning a correlation filter (CF), cyclic sampling, and fast Fourier transform. The first CF-based tracker came from Bolme et al. [19]. They designed the tracker with a minimal output sum of a squared error filter (MOSSE) using raw image pixels as inputs to train the correlation filters without any feature extraction. After Bolme, Henriques et al. [20] designed the circulant structure kernel (CSK) tracker that modeled the training of a correlation filter as a ridge regression problem, and introduced a circulant structure and kernel tricks to optimize the tracking framework. However, the CSK tracker only used greyscale features to train the filter, and this largely limited its performance. To overcome this disadvantage, Danelljan et al. [21] integrated the image‘s color properties into the CSK tracker, and experiments showed that this method significantly improved its performance. Then, with the kernelized correlation filter (KCF)/discriminative correlation filter (DCF) tracker [22] designed by Henriques et al., the process of feature extraction was further optimized. They extended the target’s feature representation into multichannel histograms of oriented gradients (HOG) and color name (CN) features instead of single-channel features, and introduced a Gaussian kernel to accelerate tracker efficiency. The long-term correlation tracker (LCT) [23] is a typical long-term tracker using CF that concentrates on solving the problem of target deformation during the tracking process, and decomposes the tracking task into two parts, translation estimation and scale estimation. The discriminative scale space tracker (DSST) [24] is another CF-based tracker that focuses on tackling the target’s fast-scale estimation. It trains a translation filter and a scale filter using handcrafted features, respectively. The background-aware correlation-filter (BACF) tracker [10] indicated that a robust tracker not only needs to boost its response to the target, but also needs to learn how to suppress its response to the background. So, this tracker utilizes background and target-variation information to build a discriminative classifier. The Staple [25] tracker uses the target‘s color-statistics information to build a histogram-score model and combine it with the model that is learned on the spatial layout of target, showing robust and balanced performance in many complex environments. The adaptive spatially-regularized correlation filter (ASRCF) [26] tracker designed a novel model which could optimize the spatial regularization weight and the filter coefficients at the same time. 

### 2.2. Deep-Learning-Based Trackers

The main characteristic of deep-learning-based trackers is that those trackers introduce a deep-learning (DL) framework into visual tracking. This characteristic can be viewed from two aspects. A deep-learning framework (especially a CNN) can be used as a powerful feature extractor. We can train a deep neural network with a large amount of data so that the network learns to extract features from different levels. Sometimes, a CNN can be integrated into a CF tracking framework as a feature-extractor component. For example, the deep spatially regularized discriminative correlation-filter (SRDCF) tracker [27] trains an CNN and employs extracted features from its shallow layers in a spatially regularized DCF tracker. The spatial temporal regularized correlation filter (STRCF) tracker [28] introduced temporal regularization into SRDCF with a single sample. The hedged deep tracker (HDT) [29] and hierarchical convolutional features (HCF) tracker [30] are also typical trackers using a CNN as feature extractor. 

The other aspect of using a deep-learning framework in visual tracking is to bring its end-to-end characteristics into full play. As discussed in Section 1, SiamFC [17] is a typical tracker of this category. This framework realized end-to-end workflow specifically for tracking problems and achieved quite competitive performance. On the basis of the SiamFC tracker, CFNet [31] integrated a correlation filter into SiamFC and interpreted the CF as a differentiable CNN layer. In this way, the CF could be trained end to end. The dynamic Siamese (DSiam) tracker [32] constructed a component which could simultaneously represent the target’s appearance and suppress background noise. The SiamRPN [33] tracker added a regression branch to the original SiamFC, and introduced a region proposal network (RPN) [34] into visual tracking. So, this tracker could largely improve its performance in target-scale estimation. DiaSiamRPN [35] tracker introduced a novel distractor-aware component on the basis of SiamRPN and improved its performance. SiamFC-tri [36] tracker designed a new triplet loss function in SiamFC to extract more powerful features. Deeper and wider SiamFC (DWSiamFC) tracker [37] analysed key factors of the CNN that influenced the tracker’s performance and showed how to make the network deeper and wider. The cascaded RPN (C-RPN) tracker [38] introduced hard negative sampling and designed the cascaded RPN to address the problem of training data imbalance. However, few trackers tried using prior information to guide the tracking process. 

## 3. Proposed Algorithm

**Figure 1:**
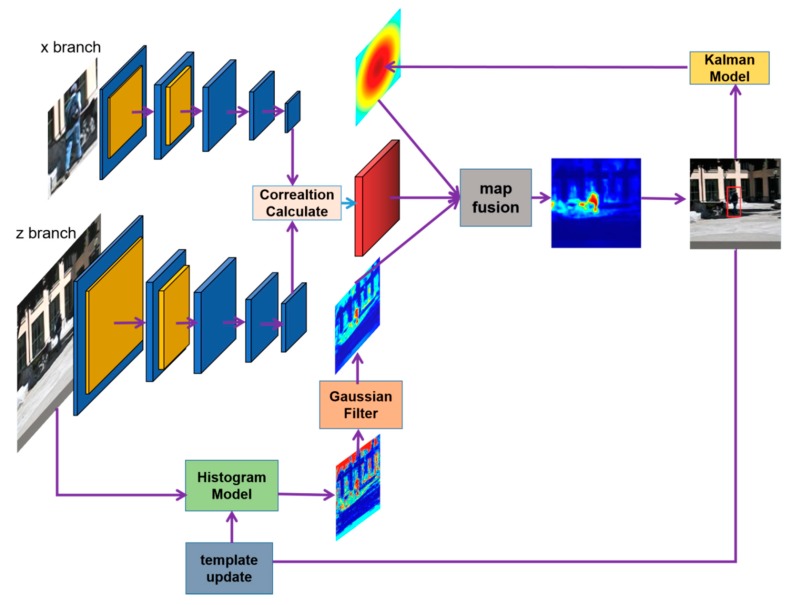
Basic workflow of our Histogram–Kalman SiamFC (HKSiamFC) tracker. The two-branch convolutional neural network (CNN) has the same architecture as the original fully-convolutional Siamese (SiamFC) tracker. We adopted the histogram model from Staple in HKSiamFC and used it to generate a histogram-score map, and we then optimized it with a Gaussian filter; this map is used to provide prior information about target appearance. We also designed a Kalman model to generate a Gaussian likelihood map based on a Kalman filter to provide prior information about target motion. Finally, we combined all three maps to capture the target.

The tracking framework we propose in this paper consists of three main components, namely, SiamFC, a histogram-score model from Staple, and a Gaussian likelihood model based on a Kalman filter. Our main purpose was to use the histogram-score and Gaussian likelihood models to suppress the background’s response score. When the SiamFC component outputs a response score map, our tracker will use the histogram score map and the Gaussian likelihood map to process the response score map by element-wise multiplication. The overview of our algorithm’s workflow is shown in Figure 1, and it is described in detail in the following sections. 

### 3.1. SiamFC Tracker

The SiamFC tracker models visual tracking as a similarity-learning problem, and trains the twin neural network to learn a general similarity-matching function. Its main architecture is made of two CNN branches that share the same parameters and are used to process the template patch and the searching-area patch, respectively. The template patch is initially set as the ground truth from the first frame. The searching-area patch is cropped from the current frame that is to be searched for the target. The template and searching-area patches are denoted as *z* and *x*, respectively. Two patches *x* and *z* were input into Alexnet and generated the feature maps denoted as φ(x) and φ(z). As the size of φ(z) was smaller, it was used as a sliding window on φ(x) to evaluate the similarity scores between z and each subregion of x. The similarity score was computed by the formulation below:

(1) $$SimiScore\left(x\left(i\right),\;z\right)=\sum_{\left(m,\;n\right)\in S}\varphi {\left(z\right)}_{\left(m,\;n\right)}\times \varphi {\left(x\left(i\right)\right)}_{\left(m,\;n\right)},\;$$

where *x*(*i*) denotes the ith subregion of *x* that had the same size as *z*, $\varphi {\left(z\right)}_{\left(m,\;n\right)}$ denotes the value vector of the pixel located at co-ordinates (*m*, *n*) on the feature maps of *z*, and *S* represents the whole area of each feature map. So, φ(z) slid onto φ(x) from the top left to the bottom right and produced a score map, and each pixel on it showed the similarity between the subregion of x and template z. Then the subregion that had the maximal value was selected as the target. 

AlexNet in SiamFC was trained by an offline pretraining method with a logistic loss function. Training data were positive and negative pairs of images. Those images were extracted from two frames of the same video that contained a target. Each pixel value of the score map used for training was labelled as +1 or −1 on the basis of its distance to the map center. If the distance were smaller than a threshold, the pixel was labelled as +1; otherwise, it was labeled as −1. The logistic loss function adopted here was defined as

(2) loss(y, r)=log(1+exp(−yr)), 

where y is the ground-truth binary score map, and *r* is the real-valued score map. So, the final loss of score map r is defined as the mean of each pixel’s loss:

(3) $$L\left(y,\;r\right)=\frac{1}{\left|D\right|}\underset{u\in D}{\sum }l\left(y\left[u\right],\;r\left[u\right]\right),\;$$

where *D* denotes the whole score map, and u∈D represents each pixel on it. AlexNet parameters were achieved by using the stochastic-gradient-descent (SGD) method. For more details about this issue, please refer to [17]. 

### 3.2. Histogram Score Model from Tracker Staple 

The key idea behind the Staple tracker is the intuition that an object’s color distribution is always stable and discriminative enough, no matter how much the object’s appearance changes. So, a model based on color statistics can provide powerful information for visual-tracking problems. The information that this model offers can be seen as prior information, and this information is what SiamFC lacks. So, we integrated this histogram-score model into HKSiamFC. 

To calculate the histogram score of an image, an N-Channel weight map is introduced; here, N is the number of input feature maps. That is to say, for each pixel on each feature map, there is a weight value provided for it to compute the histogram score. Here, this weight map is denoted as β, and this issue is formulated as

(4) $$f_{hist}\left(x;\beta \right)=h\left(\tau_{x};\beta \right),\;$$

where *x* is the original image, and $\tau_{x}$ is an N-channel feature map extracted from x, having the same channel number as β. Then, $\tau_{x}$ and β were combined to calculate the histogram score. As the histogram score should be invariant to spatial permutations of $\tau_{x}$, i.e., h(τ)=h(∏τ) should be satisfied for any permutation matrix Π, so h can be formulated as

(5) $$h\left(\tau ;\beta \right)=\beta^{T}\left(\frac{1}{\left|\varrho \right|}\sum_{u\in \varrho }\tau \left[u\right]\right),\;$$

where ϱ represents the whole area of each feature map, and τ[u] represents each pixel on ϱ. The way to compute β is to model this problem as a linear-regression problem. For any pixel that belongs to the target, the ground-truth label of $\beta^{T}\tau \left[u\right]$ should be 1, and for any pixel that belongs to the background, the ground-truth label of $\beta^{T}\tau \left[u\right]$ should be 0. Then, the loss function can be formulated as

(6) $$\frac{1}{\left|B\right|}\sum_{u\in B}{\left(\beta^{T}\tau \left[u\right]\right)}^{2}+\frac{1}{\left|O\right|}\sum_{u\in O}{\left(\beta^{T}\tau \left[u\right]-1\right)}^{2},\;$$

where O represents the area of the target region, and B represents the area of the background region. As the number of feature maps is N, and every feature map is independent of the others, this formulation can be decomposed into independent terms for each feature dimension, so that

(7) $$\sum_{j=1}^{N}\left[\frac{P^{j}\left(O\right)}{\left|O\right|}{\left(\beta^{j}-1\right)}^{2}+\frac{P^{j}\left(B\right)}{\left|B\right|}{\left(\beta^{j}\right)}^{2}\right],\;$$

where $N^{j}\left(O\right)$ denotes the number of pixels in region *O* for which feature dimension *j* is non-zero k[u]=j.Then, the solution of this regression problem is

(8) $$\beta_{t}^{j}=\frac{\rho^{j}\left(O\right)}{\rho^{j}\left(O\right)+\rho^{j}\left(B\right)+\lambda },\;j=1,\;2,\;3\;\ldots \;N,\;$$

where $\rho^{j}\left(O\right)$ is the proportion of pixels in a region for which feature *j* is non-zero. For more details about this issue, please refer to [25]. 

### 3.3. Optimize Histogram Score with Gaussian Filter

**Figure 2:**
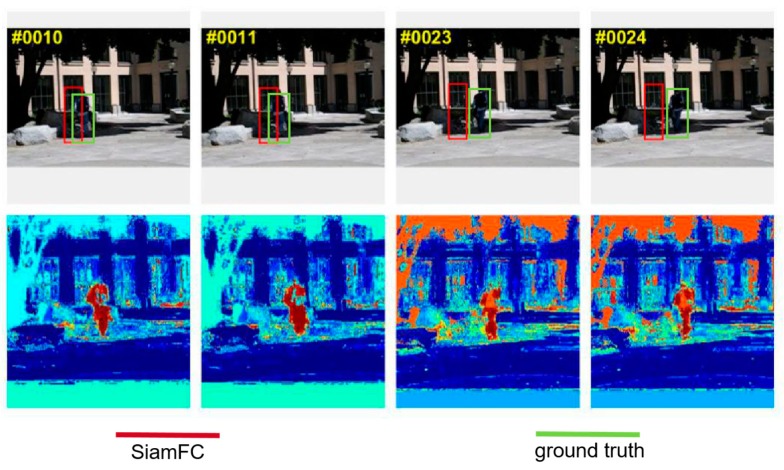
(**top**) Four frames (#0010, #0011, #0023, and #24) of SiamFC’s tracking failure in Human8 sequence; (**bottom**) histogram maps of each frame corresponding to first row (#0010, #0011, #0023, and #24), the red region in the map represents high score.

In Reference [25], weight map β was used to generate the integral map of an image. In this paper, we used β to optimize the final score map generated from SiamFC. We were inspired to do so when we saw the histogram-score map of the Human8 sequence from OTB during the experiments. As shown in Figure 2, the histogram score of the target region was larger than that of the background region (pixels having a larger histogram are marked with red). The SiamFC tracker failed badly in this sequence, even from the first few frames. The histogram-score map could have compensated for SiamFC’s failure, so we combined SiamFC’s response-score map with Staple’s histogram-score map using element-wise multiplication; this process could suppress the background-response score so that the response of the right target can be more prominent. 

As discussed in Section 3.1, the size of search-region patch x in SiamFC was 255 × 255 pixels, and the size of the final response-score map was 17 × 17 pixels. To locate the target co-ordinates, interpolation was used to resize the final response-score map to 255 × 255 pixels, the same size as the search region. In our HKSiamFC tracker, we generated another histogram-score map with a size of 255 × 255 pixels following the method introduced in [25]. However, we found that this map could not be directly used for element-wise multiplication because it contained many isolated pixels that belong to the noise but still had a higher histogram score. In Staple, the influence of these noise pixels is eliminated by generating an integral image. However, as we did not use an integral image in our method, we adopted the Gaussian filter to address this problem. 

**Figure 3:**
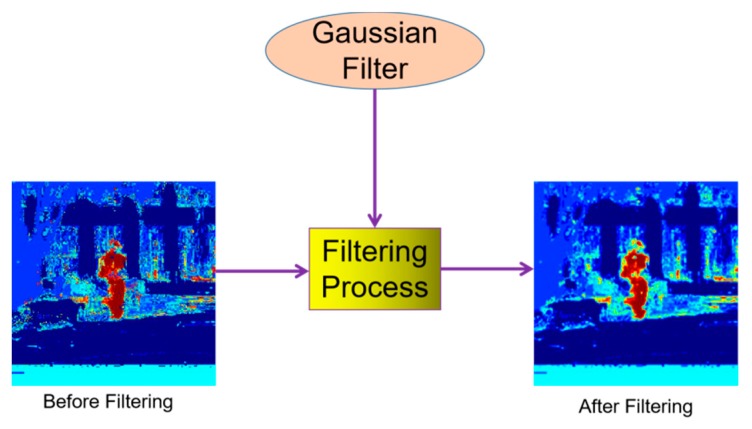
Effect of processing original histogram-score map by Gaussian filter.

A Gaussian filter is essentially a low-pass filter that calculates image convolution and normal distribution. It can remove fine image details, and produce a very pure smoothing effect. According to our experiments, in our HKSiamFC tracker, a Gaussian filter with 9 × 9 kernel size could eliminate the bad influence from the isolated noise pixels very well, as shown in Figure 3. 

As this histogram-score model was constructed on the basis of the image’s RGB color information, and experiments showed that greyscale features weaken its performance, we added a component to judge whether the image sequence was greyscale or not. The histogram model was only used when the image is in RGB color. This component made our tracker more robust. 

### 3.4. Gaussian Likelihood Map Based on Kalman Filter

Both SiamFC and Staple’s histogram model have the same disadvantage in visual tracking: they mainly concentrate on the appearance feature of the target while ignoring its trajectory trait between frames, which is also a very important indication to locate the target. When a distractor that has a very similar appearance to the target occurs, the tracker could drift to the distractor, and tracking fails. Although SiamFC has limited the search region to a small patch of the whole image, the influence of the distractor still exists. To solve this problem, we introduced a Gaussian likelihood map based on a Kalman filter. 

A Kalman filter is essentially an optimal linear-estimation algorithm used for dynamic systems. It is based on the hidden Markov model and provides a recursive solution for time-series analysis. Usually, this dynamic system can be represented by a Markov chain that contains Gaussian noise. The purpose of the Kalman filter is to predict unknown states $\left\{X_{t},\;t=1,\;2,\;3\ldots n\right\}$ from a series of measurements $\left\{Z_{t},\;t=1,\;2,\;3\ldots n\right\}$ using the following formulations:

(9) $$X_{t}=A_{t,\;t-1}X_{t-1}+W_{t},\;$$

(10) $$Z_{t}=C_{t}X_{t}+V_{t},\;$$

where A is the state-transition model, and $A_{t,\;t-1}$ denotes the transition coefficients from time t−1 to time *t*; C is the observation model, and $C_{t}$ denotes the observation at time *t*; and $W_{t}$ and $V_{t}$ are defined as the white noise at time *t* that follows Gaussian distribution. Equation (9) is called the state equation, and Equation (10) is called the measurement equation. In our proposed HKSiamFC tracker, a Kalman filter is used to estimate the target’s location co-ordinates in every frame. 

The workflow of the Kalman filter has two main steps, state prediction and measurement update. When a Kalman filter is used to estimate the target’s location, its state matrix consists of the target position co-ordinates and velocity. In the prediction step, the state equation is used to predict prior state $x_{t-1|t-1}$ and the error’s prior covariance $P_{t}$ of the *t*-th frame by

(11) $$x_{t|t-1}=F_{t-1}x_{t-1},\;$$

(12) $$P_{t|t-1}=F_{t-1}P_{t-1|t-1}F_{t-1}^{T},\;$$

where $P_{t-1|t-1}$ is the covariance of previous state $x_{t-1|t-1}$. Then, in the update step, the measurement is used to refine the state prediction. This step can be described by the formulations:

(13) $$K_{t}=P_{t|t-1}H^{T}{\left(HP_{t}H^{T}+R_{t}\right)}^{-1},\;$$

(14) $$P_{t}=P_{t|t-1}\left(I-K_{t}H\right),\;$$

(15) $$x_{t|t}=x_{t|t-1}+K_{t}\left(z_{t}-z_{t|t-1}\right),\;$$

where $K_{t}$ is the Kalman gain at time t, $P_{t}$ is the updated covariance, and I in Equation (14) denotes the identity matrix. $x_{t|t}$ is the posterior estimation of the *t*-th frame’s state. Finally, we can obtain the optimal estimation of the target’s location in the current frame from $x_{t|t}$. 

**Figure 4:**
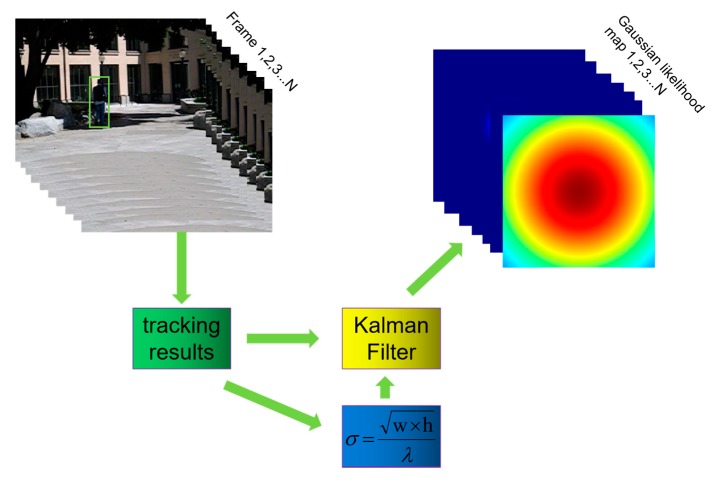
Workflow of using Kalman filter to generate Gaussian likelihood maps. The green rectangle in Frame1 is the ground-truth bounding box containing the right target.

As discussed in Section 4, neither SiamFC nor the histogram-score map has the ability to use trajectory information to distinguish targets. This means that distractors in the background can still have high response scores if they have a similar appearance to the target. After obtaining a prior estimation of the target location from the Kalman filter, this situation can be improved. We could use the estimated location as the peak to build a two-dimensional Gaussian likelihood map, and the standard deviation of this distribution is computed as

(16) $$\sigma =\frac{\sqrt{w\times h}}{\lambda },\;\lambda \in \left\{0.16,\;1.6,\;16\right\},\;$$

where *w* and *h* are the width and length of the target that are provided by the previous frame’s tracking result, respectively, while λ is a parameter. Figure 4 shows the workflow of Kalman model. This map shows the probability of each pixel on the image belonging to the target using trajectory information provided by the Kalman filter. So, utilizing it for element-wise multiplication with SiamFC’s score map can suppress background noise from the perspective of motion information. 

### 3.5. Template Update

In the course of visual tracking, the target’s appearance and its surroundings are always changing as tracking goes on, so only using the annotated ground truth in the initial frame as the template is not enough—the template needs to be properly updated over time. A template update can provide more online information about the target, and make the tracker more robust. In the HKSiamFC tracker, the update strategy is formulated as

(17) $$\{\begin{array}{c} S\left(t\right)=\left(1-R_{s}\right)\times S\left(t-1\right)+R_{s}\times S‘\left(t\right)\\ O\left(t\right)=\left(1-R_{o}\right)\times O\left(t-1\right)+R_{o}\times O‘\left(t\right)\\ B\left(t\right)=\left(1-R_{b}\right)\times B\left(t-1\right)+R_{b}\times B’\left(t\right) \end{array},\;$$

where S(t), O(t), B(t) represent the SiamFC feature template, target histogram feature template, and background histogram feature template updated in the *t-th* frame; *R* is the update coefficient. In our HKSiamFC tracker, all the update coefficients are set as 0.04.

## 4. Experiments

In this section, we evaluate the HKSiamFC tracker’s performance using the dataset of Online Object Tracking Benchmark (OTB) and Temple Color (TC128). Comprehensive experiments were performed to show the effectiveness of our tracking framework. The HKSiamFC tracker was implemented using Google’s TensorFlow library with Python. The platform we used to perform all the experiments was a Dell Alienware DESKTOP-N7K2SPB with a 3. 70 Ghz Intel Core i7-8700K CPU and an NVIDIA GeForce GTX 1080Ti GPU. The operating system was 64 bit Windows 10 Professional. Our HKSiamFC tracker can realize real-time tracking with a speed of 18 FPS. 

### 4.1. Benchmark and Evaluation Metric

OTB [39] is a famous benchmark database that was designed especially for visual tracking. It contains 100 fully labeled image sequences. The number of frames that each sequence contains range from the hundreds to the thousands. Those sequences are marked with 11 attributes according to the tracking environment, and those attributes contain illumination variation, low resolution, fast motion, background clutter, in-plane rotation, deformation, occlusion, out-of-plane rotation, motion blur, out of view, and scale variation. More concretely, the 100 sequences are divided into three subsets: OTB100 (OTB2015), OTB50, and OTB2013. OTB100 contains all those 100 sequences, while OTB50 and OTB2013 both consist of 50 video sequences that are selected from OTB100. The OTB adopts the one-pass-evaluation (OPE) protocol to evaluate tracker performance, which means that it uses the tracker to process each image sequence from beginning to end only once, and then records the tracking results. TC128 [40] is another famous dataset which is designed for visual tracking evaluation. It consists of 128 sequences of color images and those images contains all kinds of complicated tracking environments. The evaluation protocol of TC128 is the same with OTB. 

In our experiments, a uniform evaluation metric was adopted, put forward by the OTB and widely acknowledged in the community of visual tracking, namely, precision plot and success plot. These two plots are based on center location error (CLE) and intersection over union (IOU), respectively. The CLE compared the Euclidean distance between the center locations of the tracked result and the corresponding labeled ground-truth of each frame. Then, a threshold was set to determine whether tracking was successful. If Euclidean distance was smaller than the threshold, tracking was regarded as correct. Usually, different thresholds are set to more roundly evaluate trackers. The IOU is defined as

(18) $$\mathrm{IOU}=\frac{\mathrm{area}(R_{T}\cap R_{G})}{area\left(R_{T}\cup R_{G}\right)}\;,\;$$

where ∩ and ∪ denote the intersection area and union area between tracked results $R_{T}$ and labeled ground truth $R_{G}$, respectively. Thus, the precision plot shows the percentage of successfully tracked frames on the basis of CLE, and concentrates more on location co-ordinate correctness, while the success plot shows the percentage of successfully tracked frames on the basis of IOU, and concentrates more on bounding-box size. The value representing the tracker’s performance in success plot is the Area Under Curve (AUC) value. 

### 4.2. Ablation Experiment

In this section, an ablation experiment was conducted on OTB dataset to test the correctness and effectiveness of each tracking component proposed in this paper. We adopted SiamFC and Staple as our baseline trackers because our tracker was designed on that basis. These two trackers are representative state-of-the-art trackers that are cited often by visual-tracking researchers. The codes of SiamFC and Staple were downloaded from the authors’ website without any changes. The parameters of the trained CNN used in the SiamFC tracker came from “2016-08-17.net.mat” on http://www.robots.ox.ac.uk/~luca/siamese-fc.html. What’s more, to test the function of the histogram model, the Kalman model and the template update strategy of our tracking framework, we also set another three baseline trackers: (1) HSiamFC (without K): this tracker is produced by removing the Kalman model from the HKSiamFC. (2) KSiamFC (without H): this tracker is produced by removing the histogram model from the HKSiamFC. (3) HKSiamFC (without template update): this tracker is produced by removing the template update process from HKSiamFC. 

#### 4.2.1. Overall Performance

**Figure 5:**
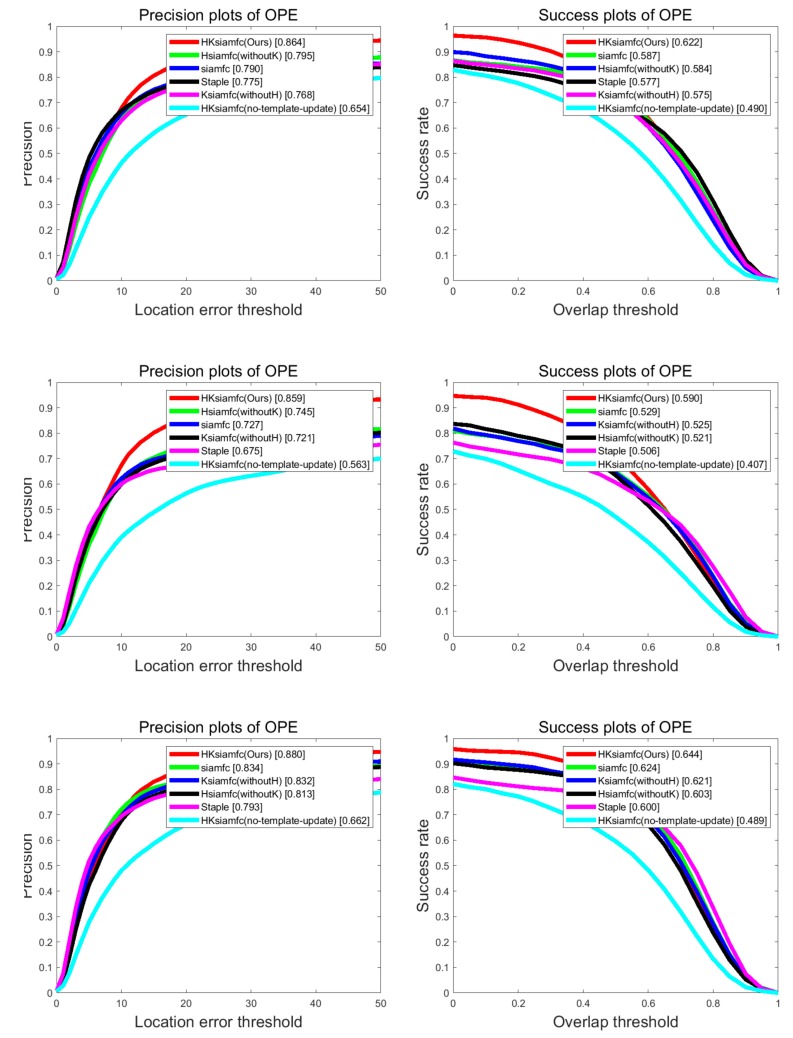
Comparison of HKSiamFC and five baseline trackers on Online Object Tracking Benchmark (OTB) dataset. Three plot pairs are results of (**top**–**bottom**) OTB100, OTB50, and OTB2013. This picture is best viewed on high-resolution displays.

Figure 5 shows the overall performance of the four trackers on the three OTB datasets in terms of precision based on CLE and success based on IOU. The HKSiamFC tracker obviously outperformed all five other trackers, no matter which dataset was used. HKSiamFC obtained average precision scores of 86.4%, 85.9% and 88.0% on OTB100, OTB50, and OTB2013, respectively. When compared with the SiamFC tracker, our HKSiamFC tracker achieved obvious gains of 7.4%, 13.2%, and 4.6% in precision. When compared with Staple, the HKSiamFC tracker achieved precision gains of 8.9%, 18.4%, and 8.7%. With regard to the success plot, HKSiamFC obtained average scores of 62.2%, 59.0%, and 64.4% on OTB100, OTB50, and OTB2013, respectively. When compared with the SiamFC tracker, our HKSiamFC tracker achieved obvious success gains of 3.5%, 6.1%, and 2.0%. When compared with Staple, the HKSiamFC tracker achieved success gains of 4.5%, 8.4%, and 4.4%. In the precision plot of OTB2013, the performance of HSiamFC (without K; 0.813) was inferior to that of SiamFC’ (0.834). The same occurred in all three success plots. As for the KSiamFC(without H) tracker, we can see that its performances are inferior to SiamFC in all the plots. So only using Kalman model can’t give a stable boost to SiamFC. What’s more, in all the six plots, the HKSiamFC(no-template-update) tracker’s performance was the worst. This situation shows the effectiveness and necessity of template update. As the appearance of the target is keeping changing, the template must be updated properly as well to keep the tracker robust. In conclusion, according to the six trackers’ overall performance on the OTB dataset, HKSiamFC showed superior performance in precision and success rate compared with its baseline trackers. Improvement was achieved because the histogram-score model could provide powerful prior information about target appearance, and the Kalman model could provide powerful prior information about target trajectory. This prior information can effectively guide the original SiamFC and compensate for its disadvantage of treating every search region equally. 

#### 4.2.2. Scenario-Based Performance

As was discussed above, image sequences in OTB are marked with 11 different labels that describe the tracking scenarios that each sequence contains. Each label represents one crucial factor that may influence tracker performance, i.e., scale variation (SV), low resolution (LR), illumination variation (IV), motion blur (MB), out-of-plane rotation (OPR), out of view (OV), background cluttered (BC), deformation (DEF), fast motion (FM), in-plane rotation (IPR), and occlusion (OCC). Evaluating tracker performance under each scenario could therefore further analyze the tracker’s characteristics and find its advantages and disadvantages. In this way, we can better optimize the tracking algorithm. 

**Figure 6:**
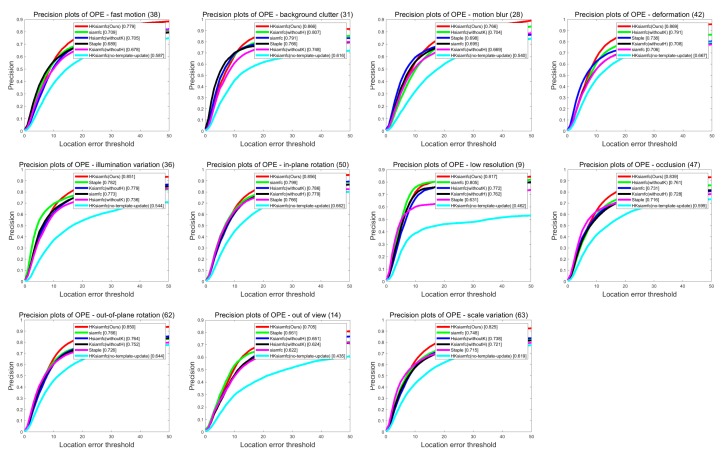
Comparison of HKSiamFC and five baseline trackers using precision-plot metric under the 11 tracking scenarios. This picture is best viewed on high-resolution displays.

**Figure 7:**
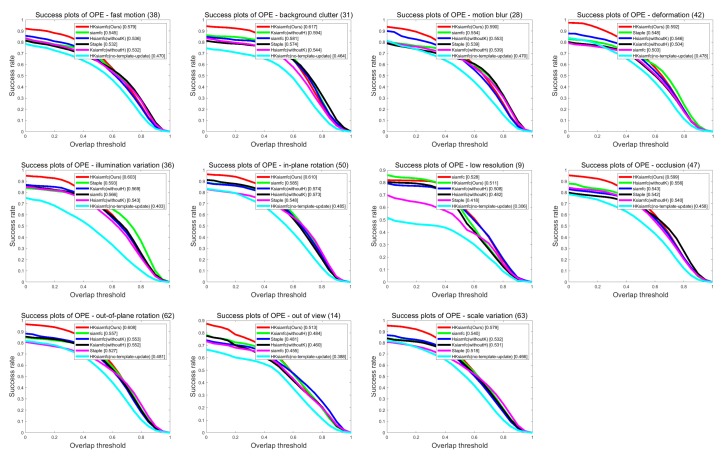
Comparison of HKSiamFC and five baseline trackers using success-plot metric under the 11 tracking scenarios. This picture is best viewed on high-resolution displays.

Figure 6 and Figure 7 show the precision plots and the success plots of the six trackers’ tracking performances in the 11 tracking scenarios on OTB100. Precision values and AUC values of the six trackers in each scenario are summarized in detail in Table 1 (performance ranking first is marked with red color). In the low-resolution sequences, HKSiamFC’s AUC value (0.511) was inferior to that of SiamFC (0.528) by gaps of 0.017. In all the other tracking scenarios, HKSiamFC’s performance was better than that of all five other baseline trackers, obviously achieving varying degrees of improvements. This proves that our tracking strategy brings comprehensive optimization to the baseline trackers. 

### 4.3. Comparison with State-of-the-Art Trackers on OTB100

To further evaluate the performance of our HKSiamFC tracker, we selected 14 state-of-the-art trackers designed on the basis of different tracking strategies as our comparison objects. Those trackers were all representative, and have been cited by many visual-tracking researchers. Those trackers are: kernelized-correlation-filter (KCF) tracker [22], multi-domain convolutional neural networks (MDNet) tracker [41], fully-convolutional Siamese (SiamFC) tracker [17], siamese network using triple loss (SiamFC_tri) tracker [36], efficient convolution operators (ECO-HC) tracker [42], context-aware deep feature compression with multiple Auto-encoders (TRACA) tracker [43], correlation filter network using triple loss (CFNet2_tri) tracker [36], cropping-inside residual fully-convolutional network (CIResNet-22FC) tracker [37], Staple tracker [25], spatially regularized discriminative correlation-filter (SRDCF) tracker [27], background-aware correlation-filter (BACF) tracker [10], discriminative correlation-filter network (DCFNet) tracker [44], large-margin correlation-filter (LMCF) tracker [45], hedged deep tracker (HDT) [29]. The selection of these trackers provided a horizontal comparison to comprehensively evaluate our HKSiamFC tracker. To be concise, we only listed the results of the precision and success plots on OTB100 because it contains all the sequences that OTB50 and OTB2013 have, so these results were persuasive enough. 

#### 4.3.1. Overall Performance

**Figure 8:**
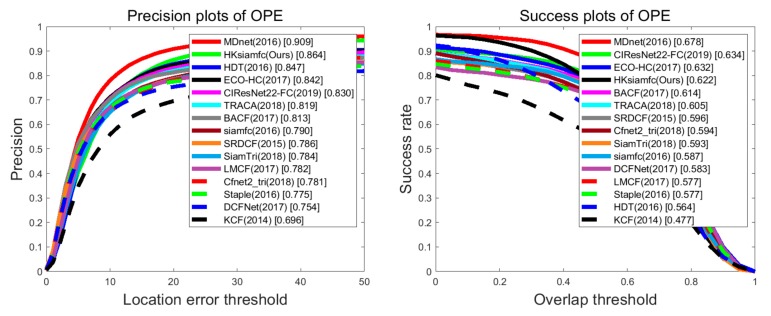
Comparison between HKSiamFC and 14 state-of-the-art trackers on OTB100. This picture is best viewed on high-resolution displays.

We compare our HKSiamFC tracker’s overall tracking performance with those of the 14 state-of-the-art trackers on OTB100. Figure 8 shows the results of the precision plot and success plot. HKSiamFC achieved the second-best performance in precision plot, only inferior to MDNet and outperforming all the other 13 state-of-the-art trackers. According to [41], the tracking speed of MDNet is 1 fps on GPU, so it is not actually a real-time tracker. HKSiamFC’s tracking speed (18 FPS) was far higher than that of MDNet. In the success plot, HKSiamFC achieved the fourth-best performance, outperforming 11 state-of-the-art trackers. So, our HKSiamFC’s overall performance on OTB100 is quite competitive within all 15 compared trackers. 

#### 4.3.2. Scenario-Based Performance on OTB100

**Figure 9:**
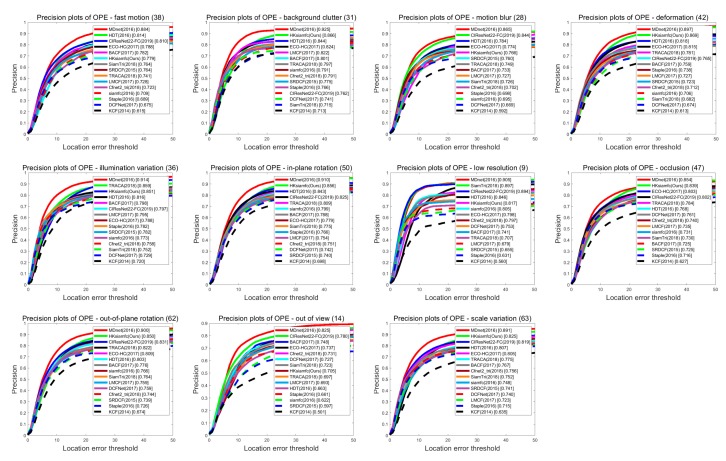
Tracking result of 15 compared trackers in precision plot in 11 different scenarios. This picture is best viewed on high-resolution displays.

We also compared HKSiamFC’s performance with that of 14 state-of-the-art trackers in 11 different scenarios on OTB100. Figure 9 shows the detailed results of all trackers’ performance in the precision plot. Table 2 shows HKSiamFC’s rankings of all scenarios in precision. We can see that HKSiamFC ranks second in six scenarios (BC, DEF, IPR, OCC, OPR, SV), more than half of the total 11 scenarios. This proves that our HKSiamFC tracker can handle many complicated tracking environments and shows competitive performance when compared with the state-of-the-art trackers. 

### 4.4. Comparison with State-of-the-Art Trackers on TC128

**Figure 10:**
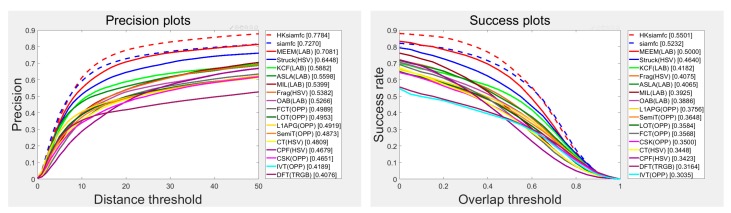
Tracking result of 18 compared trackers on Temple Color (TC128). This picture is best viewed on high-resolution displays.

We also conducted comparison experiments on the TC128 dataset. This dataset contains more image sequences than OTB100, so those experiments can show the effectiveness of our tracking strategy more comprehensively. We compared our HKSiamFC tracker’s performance with the baseline SiamFC tracker and many other classic trackers whose tracking results could be downloaded from the homepage. Figure 10 shows the precision plot and success plot of their tracking results. We can see that Our HKSiamFC tracker’s performance (0.7784, 0.5501) is obviously the best among all the trackers in both plots. 

What’s more, there are also some other state-of-the-art trackers with published average precision and success values on TC128 dataset, namely: multi-task correlation particle filter (MCPF) [46] tracker, spatially regularized discriminative correlation-filter (SRDCF) tracker [31], background-aware correlation-filter (BACF) tracker [10], hedged deep tracker (HDT) [32], convolutional networks without training (CNT) tracker [47], adaptive decontamination of spatially regularized discriminative correlation filter (SRDCFdecon) tracker [48], convolutional features for correlation filter (DeepSRDCF) tracker [49]. We also compared our HKSiamFC tracker’s performance with those trackers and details were showed in Table 3. The first and second best scores are marked with red and blue colors, respectively. We can see that HKSiamFC’s precision (77.8) ranks first in all nine trackers. HKSiamFC’s AUC value (55.1) ranks second, only inferior to MCPF by a gap of 0.1. All the experiments proves that Our HKSiamFC’s performance is still competitive on TC128 dataset. 

### 4.5. Qualitative Experiments

**Figure 11:**
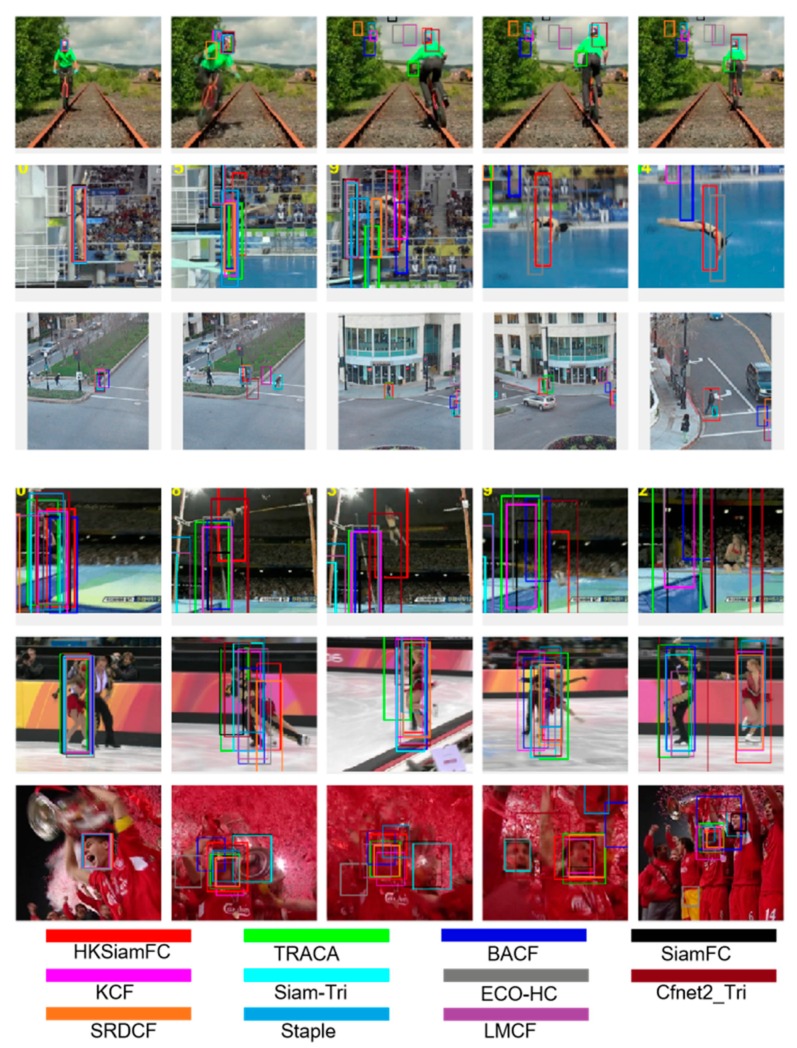
Qualitative tracking results of HKSiamFC and 10 state-of-the-art trackers on several typical sequences of OTB. Sequences (**top–bottom**) rows are Biker, Diving, Human 3, Jump, skating 2-1, and soccer. The color of each tracker is listed at the bottom of this figure.

In this section, we present the qualitative tracking results of 11 trackers listed above on several representative sequences in the OTB dataset. These 11 trackers were HKSiamFC and 10 other trackers selected from the comparison objects in Section 4.3, details of which are shown in Figure 11. The six rows of sequences (from top to bottom) are biker, diving, human3, jump, skating2-1, and soccer, respectively. 

In the Biker sequence, the most challenging moment was when the biker abruptly jumped from left to right; trackers are very easily led to drift by this fast motion before the man jumps. As is shown in the second image, some trackers already drifted into the background, and after the biker had jumped to the right, as is shown in the last three images, all other trackers drifted, while HKSiamFC is the only one that still precisely captured the target. The diving sequence is a typical sequence that contains background clutter (BC) and fast motion (FM); the audience in the background can lead the trackers to easily drift. Before the diver jumped into the river, some trackers had already failed, as is shown in the second image. While the diver was entering the river, as is shown in the fourth image, HKSiamFC was the only tracker that captured the target. In the human3 sequence, there were some other pedestrians besides the target. Those pedestrians, and even the lamppost, are powerful distractors that could mislead trackers, as is shown in the fifth image—only three trackers still captured the target, with HKSiamFC being one of them. The jump sequence is another that contains background clutters and fast motion. As is shown in the last three images of this sequence, HKSiamFC was the only tracker that captured the target while the jumper was landing. The skating2-1 sequence contains two people who constantly exchange positions and block each other, and this is the biggest challenge in this sequence. Trackers can very easily drift to the man skater, even though the woman skater is our right target. Figure 6 shows that our HKSiamFC tracker could constantly precisely capture the right target. The soccer sequence is a typical sequence of an occlusion scenario; the pieces of red paper are everywhere in the third and fourth images, and this badly influences tracker performance. We can see from the last three pictures that HKSiamFC could always capture the target, while many other trackers had already failed. 

## 5. Conclusions

In this paper, we introduced prior information of the target into the SiamFC tracker and proposed the HKSiamFC tracker. Two components were used in HKSiamFC to generate prior information: the histogram-score component from Staple to generate prior information of the target’s appearance, and the Kalman filter component to generate prior information of the target’s motion. Comparative experiments with other state-of-the-art trackers were conducted to verify the effectiveness of HKSiamFC, and results showed that HKSiamFC’s performance was quite robust in many challenging tracking environments. However, we also observed that the bounding box of the tracking result given by our tracker sometimes contained too much background information, and this could influence its precision in the success plot. So, in the future, our work will mainly focus on optimizing the tracker with regard to this issue. 

## Figures and Tables

**Table 1:** Six trackers’ average precision values and average Area Under Curve (AUC) values in 11 different scenarios.

		OCC	LR	IV	MB	OPR	OV	BC	DEF	FM	IPR	SV
**Precision**	**HKSiamFC**	0.839	0.817	0.851	0.766	0.850	0.705	0.866	0.869	0. 779	0. 856	0.825
	**SiamFC**	0.731	0.805	0.773	0.695	0.766	0.622	0.791	0. 706	0. 709	0. 799	0.748
	**Staple**	0.716	0.631	0.782	0.698	0.726	0.661	0.766	0. 783	0. 689	0. 766	0.715
	**HSiamFC**	0.761	0.772	0.736	0.704	0.764	0.624	0.748	0. 791	0. 705	0. 786	0.738
	**KSiamFC**	0.728	0.762	0.779	0.669	0.752	0.651	0.807	0. 708	0. 678	0. 779	0.721
	**HKSiamFC (No-Template-Update)**	0.599	0.462	0.544	0.540	0.644	0.435	0.616	0. 667	0. 587	0. 662	0.691
**AUC**	**HKSiamFC**	0.599	0.511	0.603	0.590	0.608	0.513	0.617	0.592	0.579	0.610	0.579
	**SiamFC**	0.543	0.528	0.566	0.554	0.557	0.455	0.581	0.503	0.545	0.585	0.540
	**Staple**	0.542	0.418	0.593	0.539	0.527	0.481	0.574	0.548	0.532	0.548	0.518
	**HSiamFC**	0.556	0.482	0.543	0.553	0.553	0.460	0.544	0.546	0.536	0.573	0.532
	**KSiamFC**	0.540	0.508	0.569	0.539	0.552	0.484	0.594	0.504	0.532	0.574	0.531
	**HKSiamFC (No-Template-Update)**	0.458	0.306	0.403	0.470	0.481	0.385	0.464	0.478	0.470	0.485	0.466

**Table 2:** HKSiamFC’s precision ranking in each scenario of all 15 trackers.

	FM	BC	MB	DEF	IV	IPR	LR	OCC	OPR	OV	SV
**Precision Ranking**	6	2	5	2	3	2	5	2	2	8	2

**Table 3:** Precision and Area Under Curve (AUC) value of the HKSiamFC and eight other trackers on the TC128 dataset(The first and second best scores are marked with red and green colors, respectively).

	HKSiamFC	MCPF	SRDCF	Deep SRDCF	Staple	BACF	SRDCFdecon	HDT	CNT
**Precision**	77.8	76.9	69.6	74.0	66.8	66.0	72.9	68.6	44.9
**AUC**	55.1	55.2	51.6	54.1	50.9	49.6	54.3	48.0	33.5
